# Prevention of malnutrition after one anastomosis gastric bypass: value of the common channel limb length

**DOI:** 10.1186/s12893-024-02438-8

**Published:** 2024-05-16

**Authors:** Elyas Mostafapour, Masoumeh Shahsavan, Shahab Shahabi Shahmiri, Noura Jawhar, Omar M. Ghanem, Mohammad Kermansaravi

**Affiliations:** 1 Minimally Invasive Surgery Research Center, Iran University of Medical Sciences, Tehran, Iran; 2 Department of Surgery, Minimally Invasive Surgery Research Center, Division of Minimally Invasive and Bariatric Surgery, Hazrat-E Fatemeh Hospital, School of Medicine, Iran University of Medical Sciences, Tehran, Iran; 3 Center of Excellence of European Branch of International Federation for Surgery of Obesity, Hazrat-E Rasool Hospital, Tehran, Iran; 4 Department of Surgery, Mayo Clinic, Rochester, MN 55902 USA

**Keywords:** Bariatric surgery, One anastomosis gastric bypass, Anemia, Hypoalbuminemia, Common channel

## Abstract

**Purpose:**

Hypoalbuminemia and anemia are commonly observed indications for one anastomosis gastric bypass (OAGB) reversal and remain significant concerns following the procedure. Sufficient common channel limb length (CCLL) is crucial to minimize nutritional complications. However, limited literature exists regarding the impact of CCLL on OAGB outcomes. This study aimed to assess the effect of CCLL on weight loss and nutritional status in patients who underwent OAGB.

**Methods:**

A prospective cohort study was conducted from August 2021 to July 2022, involving 64 patients with a body mass index of 40–50 kg/m^2^. The standardized length of the biliopancreatic limb (BPLL) for all patients in this study was set at 175 cm. Additionally, the measurement of the common channel limb length (CCLL) was performed consistently by the same surgeon for all included patients.

**Results:**

The mean age and BMI of the patients were 39.91 ± 10.03 years and 43.13 ± 2.43 kg/m^2^, respectively, at the time of surgery. There was a statistically significant negative correlation between CCLL and percent total weight loss (%TWL) at the 12-month mark after OAGB (*P* = 0.02). Hypoalbuminemia was observed in one patient (1.6%), while anemia was present in 17 patients (26.6%) at the one-year follow-up. Statistical analysis revealed no significant difference in the incidence of anemia and hypoalbuminemia between patients with CCLL < 4 m and those with CCLL ≥ 4 m.

**Conclusion:**

A CCLL of 4 m does not appear to completely prevent nutritional complications following OAGB. However, maintaining a CCLL of at least 4 m may be associated with a reduced risk of postoperative nutritional deficiencies.

## Introduction

Obesity is a prevalent chronic condition, affecting a significant number of individuals. Among the various treatment options available, metabolic and bariatric surgery (MBS) is widely recognized as the most efficacious and enduring approach to addressing obesity. Furthermore, MBS has demonstrated a remarkable safety record, with minimal postoperative complications [1, 2].

One-anastomosis gastric bypass (OAGB) is a widely accepted MBS that has attained the status of a standard treatment. It is endorsed by esteemed organizations such as the International Federation for the Surgery of Obesity and Metabolic Disorders (IFSO) and the American Society for Metabolic and Bariatric Surgery (ASMBS) [3, 4]. It was first introduced in 2001 by Rutledge [5] and later modified by Carbajo in 2005 [6]. OAGB is a relatively simple and straightforward procedure when compared to other traditional MBS bypass procedures [7]. Biliopancreatic limb length (BPLL) is an important variable to consider in OAGB cases, however, ideal BPLL remains controversial. To achieve greater weight loss, bariatric surgeons tend to choose a longer BPLL at the expense of a potentially higher rate of nutritional complications. As such, different studies have reported that complications of hypoalbuminemia and anemia tend to be the most common indications for OAGB reversal [7]. The effect of BPLL on nutritional parameters might be attributed to the available portion of absorptive surface (proportion of BPLL relative to total small bowel length (TSBL)) rather than the bypassed length. Thus, several surgeons tend to measure the TSBL and tailor the bypassed length accordingly [8]. Due to the variability in TSBL, some studies recommend at least 3 to 4 m of common channel limb length (CCLL) to be measured, rather than tailoring of BPLL, in order to prevent nutritional complications [9–11].

Due to the scarcity of literature, we aim to investigate the effect of CCLL on weight loss and nutritional status in patients who have undergone OAGB.

## Method

### Study design

This prospective cohort study was conducted on 64 patients with severe obesity (BMI of 40 to 50 kg/m^2^). We included individuals older than 18 years who underwent OAGB from August 2021 to July 2022 at the Rasool-e Akram Hospital. Additionally, all patients in this study had a follow-up period of at least one year. The study was conducted according to the Helsinki Declaration.

Patients with BMI ≥ 50 Kg/m^2^ or age < 18 years were excluded from the study. We also excluded patients who were pregnant during the follow-up period or had less than 1 year of follow-up postoperatively. As per the policy implemented in our academic metabolic and bariatric surgery center, all individuals who are considering metabolic and bariatric surgery undergo a comprehensive screening process conducted by a multi-disciplinary team (MDT). This MDT ensures that any existing anemia, as well as macro and micronutrient deficiencies, are promptly addressed and treated before the surgery. Furthermore, this dedicated MDT remains involved throughout the postoperative follow-up period, providing ongoing care and support to the patients. Following bariatric surgery, a lifelong prescription of vitamins and minerals at standardized doses is recommended for all patients to optimize their long-term well-being. The included patients had regular follow-up assessments at 1, 3, 6, and 12-month postoperative intervals by the dedicated MDT and were prescribed vitamins, minerals, Iron, and Folic acid supplements. The MDT ensured the patients’ compliance and regular use of the necessary vitamins and supplements postoperatively at each follow-up interval.

### Definitions

Total body weight loss (%TWL) was defined as = [(Initial Weight) – (Postop Weight)] / [(Initial Weight)] $\times$ 100. Change in BMI (ΔBMI) was defined as = (Initial BMI) – (Postop BMI) [12].

We considered normal measures of glucose metabolism (HbA1c < 6%, FBG < 100 mg/dL) in the absence of anti-diabetic medications as type 2 diabetes mellitus (T2DM) remission and being normotensive (BP ≤ 120/80) while off any antihypertensive medication as hypertension (HTN) remission. Additionally, a normal lipid panel or a normal lipid panel component of interest while off any lipid-lowering agent was considered dyslipidemia (DLP) remission. An AHI/RDI (apnea hypoxia index /respiratory disturbance index) of < 5 while off CPAP/BI-PAP on repeat objective testing with polysomnography (PSG) in patients previously diagnosed with obstructive sleep apnea (OSA) via a previous PSG was considered as complete OSA remission [12]. Women with a hemoglobin concentration (Hb) of < 12 g/dL, and men with Hb < 13 g/dL were classified as cases of anemia while serum albumin levels of < 3.5 g/dL were considered to be cases of hypoalbuminemia [13, 14]. A Vitamin D (25(OH) D) level of < 20 ng/ml was defined as insufficiency and normal serum calcium (Ca) levels were defined within the range of 8.5–10.5 mg/dl [15, 16].

### Surgical technique

At the time of the surgery, the patient was placed in the French position with the surgeon standing between the patient’s legs. The creation of the pouch began distal to the crow’s foot. It was fashioned by firing a transverse staple from a 45 mm endo-stapler followed by sequential vertical stapling over a 36 French calibration tube. Following our previous published studies [17, 18], the BPLL of 175 cm was uniformly established in all patients using a graded grasper. This specific length was determined based on our experiences to optimize the safety and effectiveness of OAGB. Similarly, the CCLL was measured by the same surgeon in all included patients using a graded grasper, ensuring consistency in the assessment process. A side-to-side gastrojejunostomy was created by a linear stapler, resulting in a 4 cm anastomosis at the posterior aspect of the gastric pouch.

### Data collection

Patient demographic information, baseline clinical profiles, the incidence of obesity-associated co-morbidities, and surgical data including both BPLL and CCLL were registered in the Iran National Obesity Surgery Database (INOSD) [19]. All follow-up findings were also registered and extracted from INOSD at the one-year follow-up timepoint.

### Statistical analysis

Categorical variables are presented as numbers and percentages while continuous variables are depicted as mean values with standard deviations. To explore predictors of anemia, hypoalbuminemia, Vitamin D, and calcium, a univariate regression model was used during a preliminary analysis; with one explanatory variable of clinical importance being tested in the model at a time. Subsequently, variables with significant *P* values and variables with clinical importance were further evaluated by using a binary regression model. A *P*-value of < 0.05 was considered significant for all tests. Statistical analysis was carried out with SPSS v21.0 (SPSS, Inc., Chicago, IL).

## Results

Totally 64 patients were enrolled in this study and all of them completed their one-year follow-up. The mean age and BMI were 39.91 ± 10.03 years and 43.13 ± 2.43 kg/m2, respectively, at the time of surgery. Fifty-nine (92.2%) of the patients were female. At baseline assessment, obesity-associated medical conditions included T2DM (*N* = 12, 18.8%), HTN (*N* = 11, 17.2%), DLP (*N* = 17, 26.6%) and OSA (*N* = 23, 35.9%) (Table 1). The mean length of CCLL was 579.4 ± 122.9 cm. The mean operation time was 42 ± 7.5 min. The duration of the operation was extended between 5 and 7 min due to the additional time required for measuring the common limb lengths. It is important to note that three patients were excluded from the study due to the presence of a heavy small bowel mesentery, which posed an increased risk of injury to the small bowel. We added this information to the results section.

**Table 1:** Characteristics of the patients included in the study

Variable	Mean
**Age (Year)**	39.9 ± 10.0
**Gender (F)**	59 (92.2%)
**Weight (Kg)**	114.2 ± 11.3
**BMI (Kg/m** ^**2**^ **)**	43.1 ± 2.4
**Comorbidities**	*N* = 63 (of 64 total)
**T2DM**	12 (18.8%)
**HTN**	11 (17.2%)
**DLP**	17 (26.6%)
**OSA**	23 (35.9%)

BMI: Body Mass Index, T2DM: type 2 diabetes mellitus, HTN: hypertension,

DLP: Dyslipidemia, OSA: Obstructive sleep apnea

Weight loss outcomes were reported as %TWL and subsequent BMI are shown in Table 2. Based on the obtained results, there was no significant association between CCLL and %TWL at 6 months post-op (*P* = 0.08), but there was a statistically significant negative association between CCLL and %TWL at 12 months (*P* = 0.02).

**Table 2:** Mean %TWL, weight, and BMI 6-month and 12-month post-op

Variable	6-month post-op	12-month post-op
**Weight (Kg)**	81.41 ± 9.83	72.20 ± 9.57
**BMI (Kg/m** ^**2**^ **)**	31.03 ± 3.15	27.52 ± 3.17
**%TWL**	28.71 ± 4.97	36.76 ± 5.86

BMI: Body Mass Index, %TWL: Total Weight Loss Percent

Remission and improvement of obesity-associated medical conditions one year after OAGB are shown in Table 3. For T2DM, remission was achieved in 75% of patients with a preexisting diagnosis at the 1-year follow-up. Complete HTN remission at 1-year follow-up was observed in 54.5% of HTN patients while complete remission of DLP was seen in 82.4% of patients with a preoperative diagnosis of DLP. Out of all the associated medical conditions, OSA had a 100% resolution rate at the 1-year follow-up.

**Table 3:** Remission and improvement of obesity-associated medical conditions at one year follow-up in 64 patients

Variable	12-month post-op		
Status	No change	Improved	Resolved
T2DM	1 (8.3%)	2 (16.7%)	9 (75.0%)
HTN	1(9.1%)	4 (36.4%)	6 (54.5%)
DLP	1 (5.9%)	2 (82.4%)	14 (82.4%)
OSA	0	0	23 (100.0%)

T2DM: type 2 diabetes mellitus, HTN: hypertension, DLP: Dyslipidemia,

OSA: Obstructive sleep apnea

Postoperative cases of hypoalbuminemia and anemia were found in 1 patient (1.6%) and 17 patients (26.6%) respectively, at the 1-year follow-up. Table 4 shows the association between CCLL and the incidence of anemia. The results show that there was no statistically significant difference in the incidence of anemia between patients with CCLL < 4 m and patients with CCLL ≥ 4 m (*p* = 0.942). There was also no significant difference between cases of anemia in terms of %TWL 6 and 12 months after OAGB (*P* = 0.07, *P* = 0.64). Risk factors related to the anemia were included in the univariate logistic regression analysis and showed that anemia was significantly correlated with baseline T2DM (OR [odds ratio] = 5.88, *P* = 0.010), but was not significantly correlated with any other variable (Table 5). In the multivariate analysis, the variables of T2DM, HTN, and CCLL were included. Similar to the results of the univariate analysis, the results of the multivariate analysis identified T2DM (OR = 7.704, *P* = 0.037 (Table 5).

**Table 4:** Association between common channel limb length and occurrence of anemia and hypoalbuminemia

		Common channel limb length (CCLL)		*P*-value
		**< 4 m** **(** ***n*** ** = 4)**	**≥ 4 m** **(** ***n*** ** = 60)**	
Anemia	**Yes**	1 (25.0%)	16 (26.7%)	0.942
	**No**	3 (75.5%)	44 (73.3%)	
Hypoalbuminemia	**Yes**	0	1 (1.7%)	0.795
	**No**	4 (100%)	59 (98.3%)	

**Table 5:** Binary logistic regression analysis for anemia

	Univariate			Multivariate		
Variable	***B***	OR	*P*-value	***B***	OR	*P*-value
Age	0.026	1.02	0.37	/	/	/
Common channel limb length (CCLL)	-0.002	0.99	0.48	-0.003	0.99	0.29
T2DM	1.772	5.88	0.01*	2.042	7.70	0.03*
HTN	0.044	1.04	0.95	/	/	/
DLP	0.952	2.59	0.12	-0.207	0.81	0.82
OSA	-20.275	0.01	0.99	/	/	/

T2DM: type 2 diabetes mellitus, HTN: hypertension, DLP: Dyslipidemia, OSA: Obstructive sleep apnea

When further evaluating the malabsorptive effects of OAGB, the postoperative Vitamin D, and Ca results show that there was no statistically significant difference between Vitamin D (*P* = 0.823) and Ca (*P* = 0.681) levels before and one year after OAGB (Table 6). There was also no statistically significant difference observed in Vitamin D or Ca levels in patients with CCLL < 4 m and CCLL ≥ 4 m (Table 6).

**Table 6:** Pre-operative and 12-month post-op serum vitamin D and calcium levels

	Pre-operation		12-month post-op		*P*-value
Common channel limb length (CCLL)	**< 4 m** **(** ***n*** **= 4)**	**≥ 4 m** **(** ***n*** **= 60)**	**< 4 m** **(** ***n*** **= 4)**	**≥ 4 m** **(** ***n*** **= 60)**	
Vitamin D	43.60 ± 12.01	29.63 ± 12.87	36.83 ± 7.66	38.87 ± 17.93	0.823
Calcium (Ca)	9.35 ± 0.33	9.20 ± 1.15	9.16 ± 0.44	9.27 ± 0.48	0.681

## Discussion

OAGB is a worldwide approved MBS procedure that has gradually become more popular since its introduction in 2001, primarily due to its simplicity and effectiveness. Despite the beneficial outcomes of weight loss and the improvement of obesity-related medical conditions, there are concerns related to nutritional complications following OAGB, including hypoalbuminemia and anemia [18].

The OAGB technique was initially introduced with a 200 cm BPLL, however with subsequent developments in the procedure and the emergence of nutritional complications during the post-operative period, the length was later adjusted and reduced to 150–180 cm [8]. Ahuja et al. found significantly increased incidence rates of malnutrition in OAGB patients when BPLL was extended from 150 to 250 cm [8].

Nowadays, some bariatric surgeons adjust the BPLL according to the patient’s initial preoperative BMI and the presence of preexisting obesity-related medical conditions rather than following a standard fixed BPLL [8]. The dilemma arises from the fact that a long BPLL could theoretically result in sustained weight loss, however, a longer BPLL could also lead to nutritional complications and mortality. One of the treatment choices for nutritional complications after OAGB is the lengthening of the CCLL to combat the reduced absorptive area due to an increased BPLL [9, 10, 20–22]. Concerning nutritional deficiencies such as hypoalbuminemia and anemia that typically arise in OAGB cases when compared to other MBS procedures. These nutritional complications usually occur with a BPLL equal to or greater than 200 cm. The literature reveals reports of liver failure leading to death in the context of hypoalbuminemia, in which a BPLL of 200 cm was used [8, 21–24]. In fact, in these mortality reports, the CCLL was not measured to prevent fatal complications.

Komaei et al. conducted a study in which two groups of sixty-four patients underwent OAGB, with the groups being distinguished by the BPLL used. The first group had a fixed BPLL of 200 cm while the second had a BPLL equivalent to the length of 40% of the small bowel. While there was no statistically significant difference observed in weight loss outcomes between the two groups, they did conclude that the BPLL equal to or greater than 40% of the length of the small bowel was significantly associated with a reduced rate of nutritional complications [25]. When considering which BPLL should be utilized during the OAGB procedure, it appears that a BPLL of 200 cm results in greater weight loss outcomes at the expense of nutritional complications. While a BPLL equivalent to 40% of the small bowel is successful at preventing nutritional deficiencies, it does not provide superior weight loss outcomes.

There are several recent suggestions about utilizing the CCLL as a method to prevent nutritional complications. However, there are also opinions among bariatric surgeons about the lack of a requirement to specifically measure the CCLL during the primary OAGB surgery, although some severe nutritional complications due to an insufficient CCLL have been reported [26]. Proposed strategies that have been suggested for the prevention of nutritional deficiencies, including the combination of preserving the CCLL to more than 300–400 cm and establishing a BPLL equivalent to 40% of the length of the small bowel. By following these parameters, despite a reduction in the occurrence of nutritional complications, deficiencies cannot be completely prevented as shown in the results of our study [25–28].

The cohort study conducted by Soong et al. compared two groups of patients who underwent OAGB, the first group had an unknown total length of small bowel and unknown CCLL while the second group had a measured whole length of the small bowel and approved CCLL between 400 and 600 cm. When comparing nutritional outcomes following OAGB, they reported a significantly lower incidence rate of anemia, secondary hyperparathyroidism, and hypoalbuminemia in the second group [26]. Soong et al. emphasized that measuring the whole length of the small bowel ensured a CCLL > 400 cm and implied that if a fixed BPLL of 200–250 cm was used instead, a CCLL of > 400 cm could not be attainable. This would have resulted in higher incidence rates of hypoalbuminemia and malnutrition among the patients.

According to our results, the CCLL ≥ 4 m was not significantly protective against the included nutritional complications. It is important to note that our BPLL was fixed to 175 cm in all patients. Soong et al. reported that a BPLL > 150 cm but < 200 cm allowed for a CCLL > 400 cm and maximized the metabolic effect of OAGB [8]. Within our group of 60 patients with a CCLL ≥ 4 m, there was only one case of hypoalbuminemia reported at the 1-year follow-up. In the group of 4 patients with CLL < 4 m, there were no reported cases of hypoalbuminemia. In terms of anemia, the CCLL ≥ 4 m group was found to have an incidence rate of 26.7% while the CCLL < 4 m group had an occurrence rate of 25%. Our results revealed that the incidence of anemia was not associated with the CCLL used, it is also worthwhile to mention that in all cases the ratio of the BPLL to the whole small bowel length was less than 40%. The results show that despite creating a CCLL > 4 m, nutritional deficiencies following OAGB were not avoided.

We have also identified a correlation between baseline Type 2 Diabetes Mellitus (T2DM) and anemia, which can be attributed to various mechanisms underlying anemia in T2DM patients. These mechanisms may include renal impairment leading to reduced erythropoietin levels. Additionally, factors such as oxidative stress, chronic inflammation, certain anti-diabetic medications, erythropoietin hypo-responsiveness, diabetic neuropathy, elevated levels of advanced glycation end products, and impaired erythropoietin response could contribute to anemia in individuals with T2DM [29, 30].

While it is important to acknowledge the limitations of our study, based on our findings, we cannot currently recommend routine measurement of the total small bowel length or determination of the specific CCLL during primary OAGB. To address these critical matters, further research incorporating larger patient cohorts and longer follow-up periods is necessary.

### Limitation

Our study, characterized by a relatively small sample size and a short-term follow-up period, may limit the generalizability of the findings but provides preliminary insights into the impact of common channel limb length (CCLL) on nutritional complications following OAGB. Another limitation of this study was the small sample size disparity between patients with a CCLL of less than 4 m (*n* = 4) and those with a CCLL equal to or greater than 4 m (*n* = 60). While this discrepancy could potentially impact the statistical analysis, it is important to note that this limitation arose due to the relatively short duration of the study. Another limitation was lacking a control group. Furthermore, it is important to acknowledge that there may be additional confounding factors that were not adjusted for in this study due to the disparities between the two groups. These limitations have the potential to impact the overall validity and interpretation of the results obtained. However, to gain a comprehensive understanding of this relationship, it is crucial to conduct further studies with larger sample sizes and longer follow-up durations. These future investigations will allow for more robust analysis and provide clearer insights into the role of CCLL in influencing nutritional complications after OAGB.

## Conclusions

While a CCLL of 4 m may not provide complete prevention of nutritional complications, maintaining a CCLL of at least 4 m has the potential to reduce the risk of postoperative nutritional deficiencies following OAGB. It is worth noting that a shorter CCLL demonstrates a significant correlation with short-term %TWL. However, the optimal length of the common channel and biliopancreatic limb required to fully prevent malnutrition after OAGB remains uncertain. To validate our findings and establish definitive conclusions, further cohort studies, preferably prospective in nature, with larger sample sizes and longer follow-up periods are imperative. These studies will contribute to a more comprehensive understanding of the relationship between CCLL, nutritional outcomes, and the long-term efficacy of OAGB.

## Data Availability

The datasets used during the current study are available from the corresponding author on reasonable request.
